# Myricetin Inhibits *Toxoplasma gondii* Growth, Alters Intracerebral Cyst Morphology, and Demonstrates Therapeutic Efficacy In Vivo

**DOI:** 10.3390/cells15100908

**Published:** 2026-05-15

**Authors:** Ceng-Ceng Ge, He-Xin He, Ming-Yu Pei, Shu-Qin Tang, Wei He, Man-Man Bian, Ming Pan, Si-Yang Huang

**Affiliations:** 1 Institute of Jiangsu Co-Innovation Center for Prevention and Control of Important Animal Infectious Diseases and Zoonosis, Jiangsu Key Laboratory of Zoonosis, College of Veterinary Medicine, Yangzhou University, Yangzhou 225000, China; gecengceng163@163.com (C.-C.G.); 19962611280@163.com (H.-X.H.); hsy37@163.com (M.-Y.P.); tangshuqin203@163.com (S.-Q.T.); hwhewei0707@163.com (W.H.); 17851151921@163.com (M.-M.B.); panming@yzu.edu.cn (M.P.); 2 Chongqing Academy of Animal Sciences, Chongqing 402460, China

**Keywords:** *Toxoplasma gondii*, myricetin, dihydroorotase, antiparasitic activity

## Abstract

**Highlights:**

**What are the main findings?**

- A novel anti-*Toxoplasma* (Myricetin, MYR) drug has been identified; myricetin inhibits the growth of *Toxoplasma gondii* by suppressing *Tg*DHO activity, and this growth inhibition can be partially rescued by uracil; in murine models, myricetin achieved an 80% survival rate against toxoplasmosis and induced morphological abnormalities in brain tissue cysts.

**What are the implications of the main findings?**

- This study demonstrates for the first time that myricetin effectively inhibits *T. gondii* by targeting *Tg*DHO, revealing a novel mechanism of action against the parasite. Myricetin showed a high survival benefit in an animal model and affected the morphology of brain cysts, suggesting its potential value in treating toxoplasmosis, particularly chronic infection. These findings provide an important lead compound and a new therapeutic target for the development of anti-*Toxoplasma* drugs.

**Abstract:**

*Toxoplasma gondii* (*T. gondi*) is a widespread zoonotic parasite that poses a significant threat to global public health, yet effective therapeutic options remain limited. In this study, we found that the flavonoid compound myricetin (MYR) can significantly inhibit the proliferation of *T. gondii*. This effect is associated with the inhibition of dihydroorotase (*Tg*DHO) activity in the de novo pyrimidine biosynthesis pathway, and this inhibition can be partially reversed by exogenous supplementation with uracil. Further studies revealed that MYR treatment can induce cell cycle arrest in tachyzoites and impair bradyzoite proliferation, concurrently disrupting the UDP-GlcNAc glycosylation of the cyst wall. In mouse models, MYR demonstrated significant efficacy, achieving an 80% survival rate in acute infection and inducing morphological abnormalities in intracerebral cysts during chronic infection. Collectively, these findings elucidate the anti-*Toxoplasma* activity and multifaceted mechanisms of MYR, providing valuable insights for developing novel therapeutics against toxoplasmosis.

## 1. Introduction

*Toxoplasma gondii* is an obligate intracellular apicomplexan parasite capable of infecting almost all warm-blooded animals [1,2,3]. Infection poses a particular risk to immunocompromised individuals, often leading to severe complications and even mortality [4]. Current therapeutic strategies are limited and predominantly rely on targeting the folate biosynthesis pathway with drugs such as pyrimethamine and sulfadiazine [5,6]. However, the clinical utility of these agents is constrained by their considerable toxicity and inability to clear chronic infection [7]. Given these fundamental limitations of existing therapies, the development of drugs against novel targets represents an urgent clinical need.

The survival and proliferation of *T. gondii* are highly dependent on its metabolic flexibility [8,9]. Pyrimidine nucleotide biosynthesis in the parasite is accomplished through two primary pathways: the de novo synthesis route and the salvage pathway [10]. Research indicates that the parasite relies predominantly on the de novo pathway to generate uridine-5′-monophosphate (UMP) [11], a universal precursor for all pyrimidine nucleotides and an essential substrate for RNA and DNA synthesis [12]. Disruption of key enzymes in the de novo pyrimidine biosynthesis pathway impedes parasite replication and attenuates virulence [13,14,15,16], underscoring its potential as a therapeutic target. A key distinction is that, unlike the DHO in mammals which functions as part of the multifunctional CAD complex (comprising the first three enzymes of the de novo pyrimidine synthesis pathway: carbamoyl phosphate synthetase II [CPSII], aspartate transcarbamylase [ATC], and dihydroorotase [DHO]), the *Tg*DHO operates as a monofunctional enzyme. Furthermore, *Tg*DHO shares 54.2% amino acid sequence identity with *Eimeria tenella* DHO and 37.7% with *Plasmodium falciparum* DHO, indicating that it is highly conserved among apicomplexan parasites. In contrast, its sequence identity with human DHO is much lower, ranging from 16.1% to 18.1%. In our previous work, we successfully knocked out the dihydroorotase (*Tg*DHO) gene in *T. gondii*, thereby blocking the parasite’s de novo pyrimidine synthesis. This confirmed that both PruΔ*dho* and RHΔ*dho* strains exhibited severely impaired proliferation both in vitro and in vivo. However, the growth defect in vitro could be partially rescued by the exogenous addition of uracil, indicating the limited functionality of the salvage pathway. Furthermore, we previously purified the DHO protein and validated its enzymatic activity. Mutation of the key residues H35A and D284E nearly abolished DHO activity and completely blocked parasite growth. These unique characteristics make *Tg*DHO a particularly promising drug target for toxoplasmosis [17].

Myricetin (MYR), the molecular formula is C_15_H_10_O_8_, a flavonol characterized by five hydroxyl groups (3,5,7,3′,4′,5′-hexahydroxyflavone) and plumbagin (PLU), the molecular formula is C_11_H_8_O_3_, a naphthoquinone bearing a hydroxyl group at the C5 position and a methyl group at the C2 position (5-hydroxy-2-methyl-1,4-naphthoquinone) are bioactive compounds derived from Chinese herbal medicine or other natural plants. PLU, a quinone, exhibits potent and broad-spectrum biological activities, notably strong anti-tumor and antibacterial effects. It inhibits the dihydroorotase (*Sc*DHO) from *Saccharomyces cerevisiae* [18]. Similarly, MYR inhibits viral replication via multi-target mechanisms and shows synergistic effects in models of triple-negative breast cancer and liver cancer stem cells [19]. Furthermore, MYR has been identified as a potent inhibitor of DHO in *Klebsiella pneumoniae* [19,20].

The *T. gondii* RH strain, a highly virulent type I genotype, is unable to readily form tissue cysts in the host and is therefore commonly used to establish an acute lethal infection model. In contrast, the Pru strain is a low-virulence type II genotype that can form tissue cysts in the host, making it a suitable model for studying chronic infection and the mechanism of cyst formation. The two strains correspond to the acute and chronic stages of toxoplasmosis, respectively. Therefore, in this study, the RH strain was mainly used to evaluate the anti-*Toxoplasma* efficacy of the drugs, while the Pru strain was employed to investigate the effect of the drugs on bradyzoites.

This study used *Tg*DHO as bait to screen for and identify its potential inhibitor (MYR). Subsequently, it evaluated the effects of MYR on the biological functions of *T. gondii*, elucidated the underlying mechanisms, and further investigated its therapeutic potential both in acute and chronic toxoplasmosis in mice.

## 2. Materials and Methods

### 2.1. Drug, Mice and Parasite Strains

MYR (myricetin) and PLU (plumbagin) were purchased from Solarbio Life Science, Shanghai, China. Stock solutions were prepared in dimethyl sulfoxide (DMSO) at concentrations of 500 mmol/L and 50 mmol/L, respectively, and stored at −20 °C for future use.

Seven-week-old female ICR mice (*n* = 100) were purchased from the Comparative Medicine Center of Yangzhou University and maintained under specific pathogen-free conditions, according to the guidelines specified by the Administrative Committee for Laboratory Animals of Jiangsu Province (License no. SYXK (su) 2022-0044). All animal experiments were approved by the Institutional Animal Care and Use Committee (IACUC) of Yangzhou University (permit no. 202509024, 8 September 2025). The mice were kept under standard conditions: temperature 22 ± 2 °C, relative humidity 50 ± 10%, 12 h light/dark cycle, with free access to food and water. Each cage housed up to 5 mice.

*T. gondii* type I strain RH and type II strain PRU were used in this study, and all strains were maintained in human foreskin fibroblast (HFF) cells (purchased from the ATCC, Manassas, VA, USA). HFF cells were cultured in Dulbecco’s Modified Eagle’s Medium (DMEM) supplemented with 2% fetal bovine serum (Thermo Fisher Scientific, Waltham, MA, USA), 100 U/mL penicillin, 100 μg/mL streptomycin, and 2 mM glutamine at 37 °C in a 5% CO_2_ atmosphere.

### 2.2. The Inhibitory Effects of MYR on TgDHO Catalytic Activity

The inhibitory effects of MYR or PLU on *Tg*DHO activity were determined using a spectrophotometric assay. Reactions (500 μL) contained 0.01 mg (the recombinant *Tg*DHO protein prepared in this study) of enzyme in 100 mM Tris-HCl buffer (pH 8.0), with varying concentrations of the substrate L-dihydroorotate (L-D, 1.25/2.5/5/10 mM) and in the presence or absence of MYR (1 μM) or PLU (2 μM). All assays were conducted at 37 °C for 30 min. DHO activity in the degradative direction was quantified by monitoring the decrease in absorbance of L-D at 230 nm using a NanoDrop 2000 spectrophotometer (Thermo Fisher Scientific, Waltham, MA, USA).

### 2.3. Cell Viability Assay and T. gondii Growth Inhibition Assay

The cytotoxicity of the drugs was determined in HFF cells with a CellTiter 96^®^ AQueous One Solution Cell Proliferation Assay (Promega Corp., Madison, WI, USA) as described previously [21], and the 50% cytotoxic concentrations (CC_50_) of MYR or PLU on HFF cell lines were measured with a cell proliferation assay and calculated by Prism 8. Specifically, HFF cells were seeded in 96-well plates at a density of 1 × 10^4^ cells per well and allowed to adhere overnight. The cells were then treated with various concentrations of MYR or PLU (ranging from 0.1 to 10 µM) for 48 h. Subsequently, 20 µL of CellTiter 96^®^ AQueous One Solution reagent was added to each well, and the plates were incubated for an additional 2 h at 37 °C. The absorbance was measured at 490 nm using a microplate reader. Cell viability was calculated as the percentage of absorbance relative to the DMSO-treated control group (0.1% DMSO, *v*/*v*). The RH strain expressing red fluorescent protein (RFP) was used to evaluate the inhibitory effects of compounds on *T. gondii* proliferation, and the half-maximal inhibitory concentrations (IC_50_) of the compounds against *T. gondii* growth were calculated using Prism 8 software.

### 2.4. Anti-T. gondii Activity of MYR Evaluated by a Plaque Assay

The lytic replication efficiency of the *T. gondii* RH strain was evaluated using a standard plaque assay. Confluent monolayer HFFs was cultured in 6-well plates, and 200 parasites were added to each well, treated with or without the 125 μM MYR or 250 μM uracil, incubated undisturbed at 37 °C for 7 days, then fixed with 4% paraformaldehyde (PFA), and stained with 0.2% crystal violet. All plaques were imaged under a microscope (Olympus IX73, Olympus Corporation, Tokyo, Japan), and these areas were quantified using image analysis software (Adobe Photoshop).

### 2.5. Intracellular Replication Assay

Freshly egressed RH tachyzoites were added to HFF monolayers and allowed to infect for 2 h, after which extracellular parasites were removed by washing with PBS. The medium was then replaced with fresh medium containing either 125 μM MYR, 250 μM uracil, or no addition (control), followed by incubation at 37 °C under 5% CO_2_ for 12 h. After incubation, 100 parasitophorous vacuoles (PVs) were randomly selected for observation, and the number of parasites in each PV was counted. All data presented represent the mean ± SD from three independent biological replicates. Statistical analysis was performed using GraphPad Prism 8.0.

### 2.6. Proportions of T. gondii Tachyzoites at Three Replication Stages

Freshly egressed *T. gondii* RH tachyzoites were allowed to invade HFF monolayers for 2 h at 37 °C under 5% CO_2_. Extracellular parasites were subsequently removed by PBS and then incubated for 12 h in fresh medium with or without MYR. Cells were fixed with 4% PFA and immunostained with anti-IMC1 primary antibody followed by Alexa Fluor^TM^ 488-conjugated goat anti-rabbit IgG secondary antibody (1:1000; Invitrogen, Cat# A11008, Carlsbad, CA, USA). Replication progression was classified into three morphologically distinct stages: before budding, budding, and cytokinesis.

### 2.7. Bradyzoite Differentiation Assay and Cyst Wall Analysis

HFF monolayers grown on glass coverslips were infected with PRU tachyzoites for 1 h at 37 °C under 5% CO_2_. Extracellular parasites were then removed by PBS, and infected cells were maintained in alkaline medium (RPMI 1640 supplemented with 25 mM HEPES, pH 8.2) with or without MYR to induce stage conversion from tachyzoites to bradyzoites. Cultures were incubated in a sealed, CO_2_-free environment at 37 °C for several days, with medium replacement every 24 h to sustain alkaline conditions. The conversion efficiency and bradyzoite replication were evaluated by an immunofluorescence assay (IFA) using the following markers: a rabbit anti-IMC1 polyclonal antibody for detecting the parasite cytoskeleton and a dual cyst-wall labeling approach consisting of either FITC-conjugated *Dolichos biflorus agglutinin* (DBA; 1:1000, Vector Laboratories, Cat# FL-1031, Newark, CA, USA) or biotinylated succinylated wheat germ agglutinin (s-WGA; 1:1000, Vector Laboratories, Cat# B-1025, Newark, CA, USA) followed by streptavidin-conjugated Alexa Fluor™ 594 (1:1000, Invitrogen, Cat# S-11227, Carlsbad, CA, USA). Parasitophorous vacuoles (PVs) positive for DBA or s-WGA in each group were counted. Data are presented as the mean ± SD from three independent biological replicates.

### 2.8. Effect of MYR on Acute Toxoplasmosis in Mice

Mice were randomly allocated into two groups (*n* = 10 per group) and intraperitoneally inoculated with 100 freshly egressed RH tachyzoites. At 24 h post-infection, each mouse received a daily intraperitoneal injection of either MYR (dissolved in sterile saline containing 5% DMSO, in a total volume of 50 μL) or the vehicle control (sterile saline containing 5% DMSO, in a total volume of 50 μL). Survival and body weight were recorded daily for 30 consecutive days. The appearance of obvious ruffled fur and increased ascites suggested successful infection, and microscopic examination of ascites from dead mice further confirmed the infection. Statistical analysis and generation of survival curves were performed using GraphPad Prism 8.0.

### 2.9. Effect of MYR During the Late Acute Through Chronic Phases in Mice

Mice were randomly divided into two groups (*n* = 5 per group), and each mouse was intraperitoneally inoculated with 2 × 10^5^ PRU tachyzoites. Starting on day 15 post-infection, mice received daily intraperitoneal injections of either MYR (dissolved in sterile saline containing 5% DMSO, in a total volume of 50 μL) or the vehicle control (sterile saline containing 5% DMSO, in a total volume of 50 μL). At day 28, mice were euthanized by carbon dioxide (CO_2_) inhalation followed by cervical dislocation. Brain tissues were immediately harvested, and each whole brain was homogenized individually in 1 mL of phosphate-buffered saline (PBS). The brain tissue cysts were stained with DBA and WGA, followed by quantitative cyst counting. Statistical analysis was performed, and graphs were generated using GraphPad Prism 8.0.

### 2.10. Statistical Analysis

All statistical analyses were performed in GraphPad Prism 8.0 using a Student’s *t* test, a one or two-way analysis of variance (ANOVA), or a logrank Mantel–Cox test, as indicated in the figure legends. Statistical data are expressed as the mean value ± SD of data from at least three independent experiments.

## 3. Results

### 3.1. Both MYR and PLU Exhibit a Strong Inhibitory Effect on TgDHO Activity

**Figure 1:**
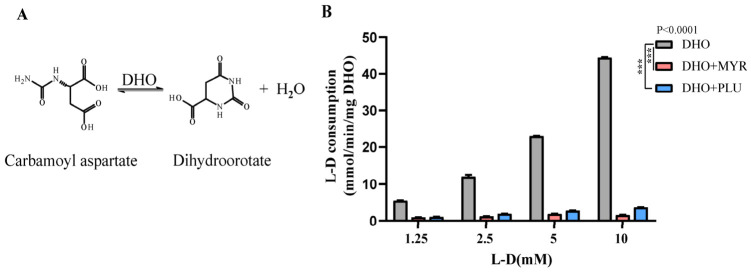
MYR inhibits DHO enzyme activity. (**A**) Catalytic mode diagram: dihydroorotase (DHO) catalyzes the conversion of carbamoyl aspartate to dihydroorotate. (**B**) Effect of MYR and PLU on the activity of *Tg*DHO enzyme. Data are presented as the mean ± SD (*n* = 3 independent experiments); *** *p* < 0.001, by Student’s *t*-test.

Myricetin (MYR) has been identified as a potential *Kp*DHO inhibitor in *Klebsiella pneumoniae*, while plumbagin (PLU) potently inhibits *Sc*DHO activity in *Saccharomyces cerevisiae* [18,20]. To evaluate the impact of MYR and PLU on *T. gondii*, we examined their effects on *Tg*DHO enzymatic activity. *Tg*DHO catalyzes the reversible interconversion between carbamoyl aspartate and dihydroorotate (Figure 1A). Across a range of L-D concentrations, *Tg*DHO activity was strongly inhibited by both MYR and PLU (Figure 1B). Therefore, MYR and PLU are potential inhibitors of *Tg*DHO.

### 3.2. MYR Inhibits T. gondii Proliferation

**Figure 2:**
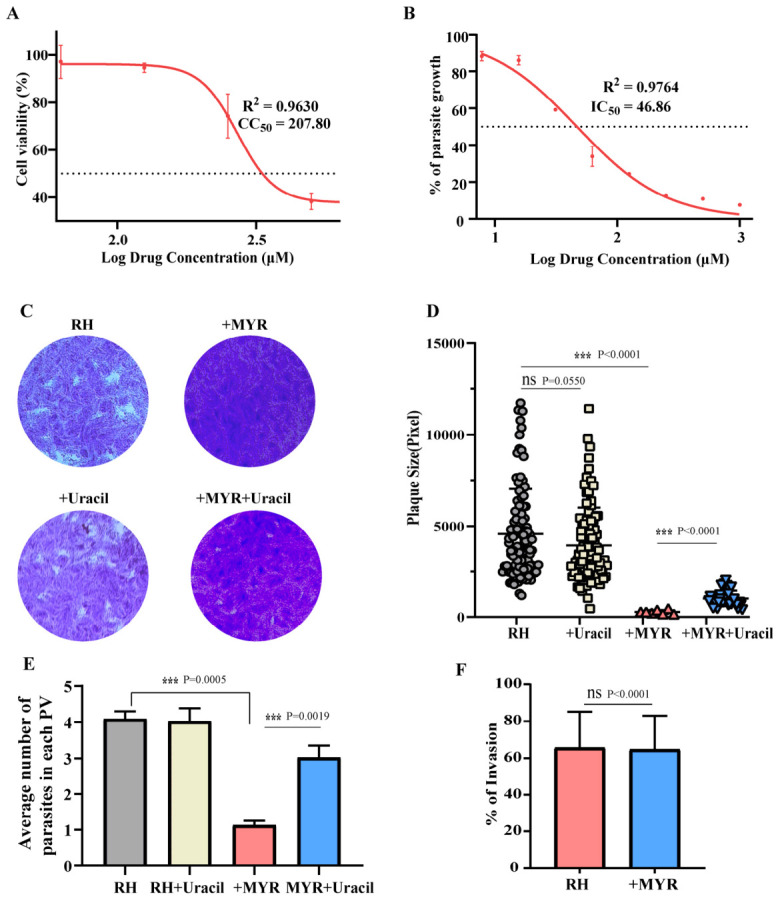
MYR inhibits the proliferation of *T. gondii*. (**A**,**B**) IC_50_ and CC_50_ of MYR on RH in vitro. (**C**,**D**) Plaques formed by *T. gondii* over 7 days. Data are presented as the mean ± SD (the dotted line represents 50%, *n* = 3 independent experiments); *** *p* < 0.001; ns, not significant; by one-way ANOVA. (**E**) Replication of *T. gondii* within 12 h under treatment with or without MYR. Data are presented as the mean ± SD (*n* = 3 independent experiments); *** *p* < 0.001; by Student’s *t*-test. (**F**) Invasion of *T. gondii* under treatment with or without MYR. ns, not significant; by Student’s *t*-test.

**Figure A1:**
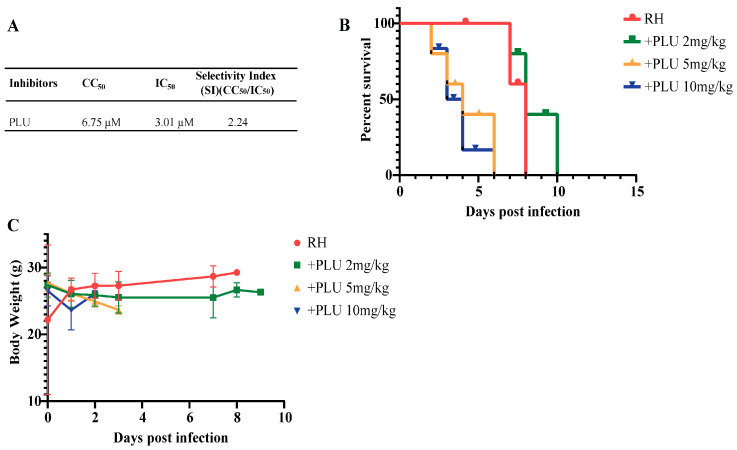
(**A**) Determination of the IC_50_ and CC_50_ of PLU. (**B**,**C**) Therapeutic efficacy of PLU in a murine model of acute toxoplasmosis. Mice were intraperitoneally inoculated with 100 RH strain tachyzoites, followed by intraperitoneal administration of different concentrations of PLU 24 h post-infection. Mouse survival (**B**) and body weight changes (**C**) were monitored.

To evaluate the effects of MYR and PLU on *T. gondii* growth, the half-maximal cytotoxic concentration (CC_50_) of each drug on HFFs and the half-maximal inhibitory concentration (IC_50_) against *T. gondii* RH tachyzoites were determined (Figure 2A,B and Figure A1A). Both drugs demonstrated an inhibitory effect on parasite growth, while, unfortunately PLU exhibited a marked cytotoxicity (Figure A1A). Therefore, MYR was chosen for subsequent studies.

**Figure A2:**
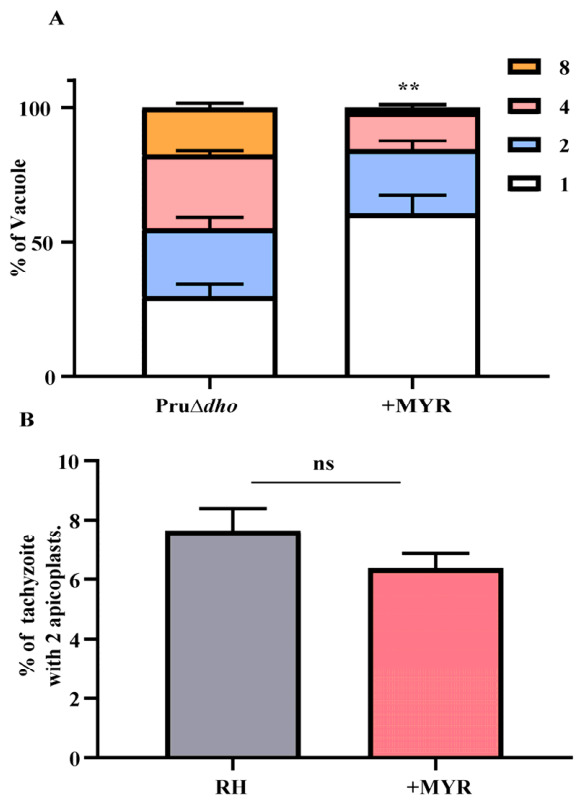
(**A**) Average number of parasites per PV in *T. gondii* Pru and Pru∆*dho* tachyzoites at 12 h post-invasion. Data are presented as mean ± SD of three independent experiments. Statistical analysis was performed by two-way ANOVA, ** *p* < 0.01. (**B**) The proportion of non-budding tachyzoites with 2 apicoplasts, ns, not significant.

To further investigate the impact of MYR on *T. gondii*, RH tachyzoites were cultured for 7 days in the presence or absence of 125 μM MYR, with or without 250 μM uracil supplementation, after which plaque formation was assessed. Under MYR treatment, RH tachyzoites failed to form normal plaques. However, this defect was partially reversed by the addition of uracil. Moreover, when parasites were treated with 125 μM MYR for 2 h post-invasion, the average number of tachyzoites per parasitophorous vacuole (PV) was significantly reduced compared to the untreated control (which typically contained approximately four tachyzoites per vacuole) (Figure 2E), demonstrating that MYR inhibits the intracellular replication of tachyzoites. However, uracil supplementation only partially rescued the replication defect. To investigate whether MYR has additional targets and to assess its specificity for *Tg*DHO, we applied MYR to DHO-deficient parasites. MYR retained inhibitory effects on the proliferation of PruΔ*dho* (Figure A2A), indicating its multi-target nature, which is consistent with previous reports. Additionally, following a 2-h invasion period of RH tachyzoites into HFFs, no significant difference in the invasion rate was observed across the tested concentrations of MYR (Figure 2F). This result suggests that the inhibitory effect of MYR is against the replicative stage rather than the invasion process. Together, these results show that MYR impairs tachyzoite proliferation, a defect that can be partially rescued by uracil.

### 3.3. MYR Induces Cell Cycle Arrest and Aberrant Replication in T. gondii

**Figure 3:**
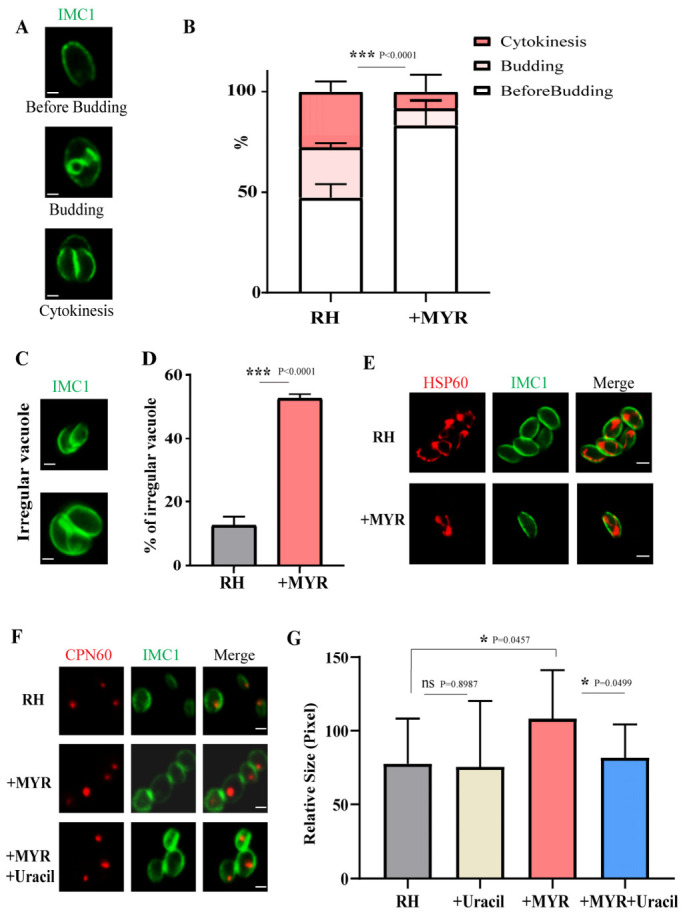
MYR induces cell cycle arrest and aberrant replication in *T. gondii*. (**A**) Three typical stages of *T. gondii* division: before budding stage: no budding observed in the inner membrane complex (IMC) of the mother parasite. Budding stage: two daughter IMCs form within the mother parasite IMC. Cytokinesis stage: daughter parasites undergo binary fission. Bar = 3 μm. (**B**) Assessment of division in *T. gondii* RH tachyzoites treated with MYR or solvent control (DMSO) for 12 h. Data are presented as the mean ± SD (*n* = 3 independent experiments); *** *p* < 0.001; by two-way ANOVA. (**C**) Abnormal replication vacuole of *T. gondii* induced by MYR treatment. Bar = 3 μm. (**D**) Statistical analysis of the proportion of *Toxoplasma* exhibiting abnormal division in the MYR-treated group. Abnormal types include asynchronous multiplication, abnormally enlarged nuclear volume, unequal progeny distribution, and defective inner membrane budding. Data are presented as the mean ± SD (*n* = 3 independent experiments); *** *p* < 0.001, by Student’s *t*-test. (**E**) Mitochondrial morphology in RH tachyzoites after 12 h of replication. (**F**,**G**) Apicoplast morphology (**F**) and size quantification (**G**) in RH tachyzoites after 12 h of replication. Data are presented as the mean ± SD (*n* = 3 independent experiments); Bar = 3 μm. * *p* < 0.001, by Student’s *t*-test.

To further investigate the inhibiting mechanism of MYR on *T. gondii*, inner membrane complex protein 1 (IMC1) was used to categorized the early cell cycle stages (before budding, budding, and cytokinesis) (Figure 3A). From the results, we found that MYR induced tachyzoite arrest at the before budding stage (Figure 3B), and tachyzoites exhibited significant replication abnormalities in the MYR-treated group, including asynchronous proliferation, unequal progeny partitioning, and defective budding morphology (Figure 3C,D).

To investigate the effect of MYR on *Toxoplasma* subcellular organelles, mitochondria and apicoplasts were stained, and the results showed no significant alterations in mitochondrial morphology upon MYR treatment (Figure 3E). In contrast, drug-exposed tachyzoites exhibited a rounded morphology, phenotypic characteristics consistent with S-phase progression. Notably, apicoplast duplication in *Toxoplasma* occurs during the S-phase and culminates in binary fission by the late S-phase. To test this hypothesis, we quantified the proportion of non-budding tachyzoites with 2 apicoplasts (Figure A2B), but no significant difference was observed between MYR-treated and control groups. However, although the proportion showed no statistical difference, the projected area of the apicoplast was significantly larger in MYR-treated parasites compared to controls. This observation aligns with the phenomenon of organelle expansion during the S-phase, and the difference could be partially restored by uracil supplementation (Figure 3F,G). As the S-phase represents the critical DNA synthesis window regulated by pyrimidine metabolism, our previous work revealed that DHO deficiency reduces uridine monophosphate (UMP) levels—the central precursor for pyrimidine nucleotide biosynthesis and DNA replication. However, due to the inability to collect sufficient parasite biomass for UMP measurement under MYR treatment, we could only speculate that MYR may delay S-phase DNA synthesis by inhibiting *Tg*DHO activity and consequently depleting nucleotide substrates. Collectively, these findings indicate that MYR induces both cell division progression arrest and abnormal replication in *T. gondii*.

### 3.4. MYR Disrupts the Glycosylation of the Cyst Wall

**Figure 4:**
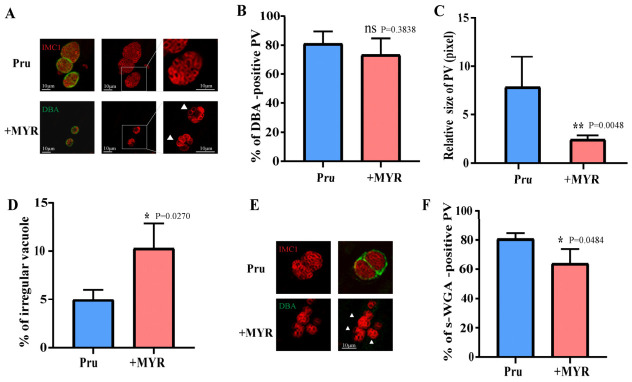
Effects of MYR on *T. gondii* bradyzoites. (**A**) Immunofluorescence staining of Pru strain parasites after 5 days of differentiation in alkaline RPMI 1640 medium. Red: IMC1 (parasite marker); Green: DBA (cyst wall marker). White arrows indicate bradyzoites exhibiting abnormal replication in the MYR-treated group. Bar = 10 μm. (**B**) Proportion of DBA-positive cysts in DMSO- or MYR-treated groups. Data are presented as the mean ± SD (*n* = 3 independent experiments); not significant (ns, *p* > 0.05, by Student’s *t*-test). (**C**) Size (relative pixel area) of DBA-positive cysts in DMSO- or MYR-treated groups. Data are presented as the mean ± SD (*n* = 3 independent experiments); ** *p* < 0.01, by Student’s *t*-test. (**D**) Proportion of cysts exhibiting abnormal replication (as indicated by arrows in (**A**)) in MYR-treated groups. Data are presented as the mean ± SD (*n* = 3 independent experiments); * *p* < 0.05, by Student’s *t*-test. (**E**,**F**) Pru strain parasites after 3 days of differentiation in alkaline RPMI 1640 medium: Representative immunofluorescence images (**E**) and quantification of s-WGA-positive cysts (**F**). Red: IMC1 (parasite marker); Green: s-WGA (cyst wall marker). Bar = 10 μm. Data are presented as mean ± SD (*n* = 3 independent experiments); * *p* < 0.05, by Student’s *t*-test.

To investigate the impact of MYR on bradyzoite development, glycosylation of the cyst wall was analyzed by staining lectin. *Dolichos biflorus agglutinin* (DBA, which binds to GalNAc residues), succinylated wheat germ agglutinin (s-WGA, which recognizes GlcNAc and its oligomers) and its oligomers were employed to assess specific glycosylation patterns [22,23]. From the results, we found that there was no significant difference in the DBA-positive rate of cyst walls between treated and untreated groups (Figure 4A,B). Additionally, bradyzoite size was quantified to assess the impact of MYR on bradyzoite replication. Like its effect on tachyzoites, MYR treatment partially inhibited bradyzoite replication (Figure 4C). Furthermore, MYR induced abnormal division events in bradyzoites, mirroring the phenotype observed in tachyzoites (Figure 4D). In conclusion, MYR not only impedes bradyzoite replication but also disrupts GlcNAc glycosylation in the bradyzoite cyst wall.

The s-WGA-positive rate was significantly reduced in the treated group (Figure 4E,F). These results indicate impaired UDP-GlcNAc-dependent glycosylation in the cyst wall and phenocopying strains with deletion of the *dho* gene involved in pyrimidine de novo synthesis pathway. This glycosylation defect likely stems from inhibition of the pyrimidine synthesis pathway by MYR. Reduced UMP synthesis consequently diminishes the cellular pool of UDP-sugar precursors, including UDP-GlcNAc. The dependence of GlcNAc synthesis in *T. gondii* on the GNA1 acetylation step, an enzyme highly sensitive to UDP precursor availability, provides a plausible mechanistic explanation for the observed s-WGA reduction.

### 3.5. MYR Exhibits Anti-Toxoplasmosis Activity in Mice

**Figure 5:**
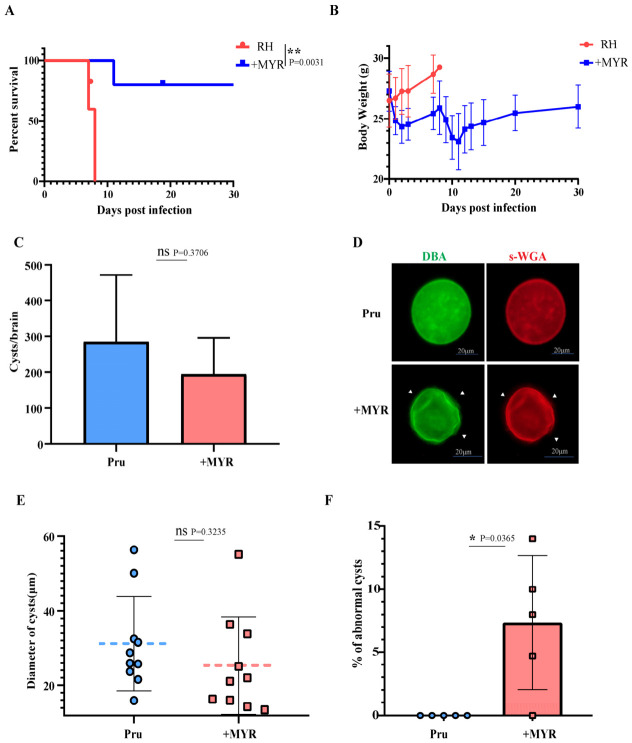
Evaluation of MYR efficacy against *T. gondii* infection in vivo. (**A**,**B**) Mice were intraperitoneally (i.p.) injected with 100 RH tachyzoites, followed by i.p. administration of 30 mg/kg MYR at 24 h post-infection. Survival rate (**A**) and body weight changes (**B**) were monitored; *n* = 10 mice, ** *p* < 0.01, by log-rank Mantel-Cox test. (**C**–**F**) Mice were i.p. injected with 10^5^ Pru tachyzoites. At 15 days post-infection, 30 mg/kg MYR was administered i.p. Brain tissues were collected at day 28 for quantification of (**C**) cyst burden (ns, not significant; by Student’s *t*-test.) and (**D**) brain cyst morphology (white arrows denote abnormal brain cyst morphology) (**E**) and cyst diameter (the blue and red dashed lines indicate the average value, ns, not significant; by two-way ANOVA). (**F**) The proportion of cysts exhibiting abnormal morphology was quantified; * *p* < 0.05, by Student’s *t*-test.

To evaluate the therapeutic potential of MYR against acute toxoplasmosis in vivo, an acute toxoplasmosis model of mice was used. The results showed that MYR treatment achieved an 80% survival rate with gradual recovery of body weight (Figure 5A,B). Although PLU exhibited cytotoxicity in vitro, the possibility of its therapeutic efficacy against toxoplasmosis in vivo was not excluded in this study. Therefore, various concentrations of PLU were administered to treat acute toxoplasmosis. However, none of the doses showed significant therapeutic efficacy, as all mice died within 15 days (Figure A1B,C).

To explore the potential efficacy of MYR against toxoplasmosis during the late acute through chronic phases, mice were inoculated intraperitoneally (i.p.) with 10^5^ Pru strain tachyzoites. MYR or equal-volume DMSO (solvent control) treatment was initiated 15 days post-infection. Brain tissues were harvested on day 28 i.p. for quantitative analysis of brain cysts. Although a decreasing trend in cyst burden was observed in MYR-treated mice compared to controls, the difference did not reach statistical significance (*p* > 0.05). Simultaneously, the mean cyst diameter in the MYR group was 25.36 ± 13.06 μm (mean ± SD) compared to 31.19 ± 12.63 μm (mean ± SD) in the control group; this difference was also statistically non-significant (*p* > 0.05). Further assessment of cyst wall glycosylation status using DBA and s-WGA staining revealed that MYR treatment did not significantly alter the glycosylation pattern (Figure 5A–E). This suggests that *T. gondii* may accumulate sufficient sugar nucleotide precursors (e.g., UDP-GlcNAc/UDP-GalNAc) during the initial infection phase (the pre-treatment 15 days) to sustain cyst wall formation. Notably, aberrant brain cysts (non-spherical/irregular) were observed in the MYR treatment group, constituting 7.34 ± 5.308% (mean ± SD) of the total cysts (Figure 5F). This indicated that MYR did not significantly suppress the overall cyst number or size, while it might interfere with normal cyst development. In summary, MYR not only exerts therapeutic effects against early acute toxoplasmosis but also induces morphological abnormalities in brain cysts during the late acute to chronic phases of infection.

## 4. Discussion

MYR, a naturally occurring flavonoid widely present in fruits, vegetables, and tea leaves, has been demonstrated in this study to effectively inhibit *Tg*DHO enzymatic activity and exhibit anti-*Toxoplasma* effects in mice models. Specifically, MYR treatment delayed the parasite cell division progression and disrupted UDP-GlcNAc glycosylation on the cyst wall of bradyzoites. We speculate that these phenotypic alterations may align with metabolic disturbances resulting from decreased UMP levels: UMP depletion reduces the supply of UDP-GlcNAc precursors, thereby impairing cyst wall glycosylation.

MYR and PLU have been shown to inhibit DHO activity in other species. Both compounds have been identified as multi-target agents with broad-spectrum potential. *Tg*DHO catalyzes the synthesis of dihydroorotate, and both MYR and PLU strongly inhibited its enzymatic activity (Figure 1B,C). Due to the apparent toxicity of PLU (Figure A1A), it was not studied in depth; nevertheless, we evaluated its therapeutic potential against toxoplasmosis in mice. However, PLU still failed to show satisfactory treatment efficacy (Figure A1B,C).

MYR exhibited a CC_50_ of 207.8 μM in HFFs and an IC_50_ of 46.86 μM against *T. gondii* (Figure 2A,B). MYR also carries a toxicity risk, and subsequent work will focus on identifying its structural analogs to develop safer and more effective anti-toxoplasmosis agents. MYR significantly reduced plaque formation (Figure 2C,D) and inhibited *T. gondii* replication in vitro (Figure 2E), and this inhibition could be partially rescued by exogenous uracil supplementation, confirming that MYR inhibits *Tg*DHO [24,25,26,27].

Further analysis revealed that MYR treatment arrested parasites at the pre-budding stage (Figure 3A,B) and induced apicoplast enlargement along with a rounded morphological phenotype (Figure 3F,G), resembling S-phase delay, which likely results from nucleotide shortage due to UMP deficiency. During the S phase of mammalian cells, a substantial supply of pyrimidine nucleotides is required for DNA replication [28]. Increased activity of CAD, a multifunctional protein encoding carbamoyl-phosphate synthetase II, aspartate transcarbamylase, and dihydroorotase (CPSII/ATC/DHO), ensures the sufficient production of pyrimidine nucleotides, thereby supporting uninterrupted DNA synthesis. Inadequate provision of pyrimidine nucleotides impedes DNA replication and can lead to cell cycle arrest at the S-phase checkpoint [27]. We therefore speculate that MYR inhibits DHO activity, depleting UMP pools and consequently prolonging DNA replication duration, ultimately triggering S-phase arrest. Regrettably, insufficient parasite yield post-MYR treatment precluded direct UMP quantification to validate this model. Collectively, these data demonstrate that MYR disrupts *Toxoplasma* cell cycle progression through the induction of S-phase arrest.

In addition to tachyzoites, MYR also inhibited bradyzoites replication (Figure 4C,D). Although no significant change was observed in DBA staining positivity, s-WGA signal was markedly reduced, reflecting impaired UDP-GlcNAc-dependent glycosylation. Recent studies have shown that GlcNAc synthesis in *T. gondii* depends on the GNA1 acetyltransferase, whose activity is highly sensitive to UDP precursor concentration—a finding consistent with our observations. Glycosylation modifications on the cyst wall of *T. gondii* bradyzoites are crucial for its structural integrity, immune evasion, and the persistence of parasitic infection [23,29]. Lectins such as (DBA) and s-WGA are commonly used to detect and study glycosylation patterns [30]. DBA primarily recognizes α-or β-linked N-acetylgalactosamine (GalNAc), while s-WGA mainly binds to N-acetylglucosamine (GlcNAc). Furthermore, antibodies or lectins targeting the glycosylation modifications of the cyst wall may also be utilized for diagnostic and therapeutic purposes [31].

In mouse models, MYR demonstrated therapeutic efficacy against acute infection (Figure 5A,B). During the late acute through chronic phases, although administration initiated at 15 days post-infection for 13 days did not significantly reduce brain cyst burden, abnormal cyst morphology (Figure 5D,F) was observed, indicating a biological effect of the drug, albeit limited by the treatment window and duration.

This study has the following limitations: first, myricetin exhibits good inhibitory effects against *T. gondii* both in vitro and in vivo; however, its selectivity index is suboptimal, and the toxicity issue cannot be overlooked. Second, the inhibitory effect of myricetin on *Tg*DHO is not specific; therefore, no specific targeted inhibitor of *Tg*DHO has been identified in this study.

## 5. Conclusions

Our findings demonstrated that MYR inhibited *Tg*DHO enzyme activity, exerts significant anti-*Toxoplasma* effects both in vitro and in vivo, and induces morphological abnormalities in brain cysts. These results provide new insights and experimental evidence for the development of therapeutics drugs both for acute and chronic toxoplasmosis.

## Data Availability

The original contributions presented in this study are included in the article. Further inquiries can be directed to the corresponding author.
